# SUN-Family Protein *UvSUN1* Regulates the Development and Virulence of *Ustilaginoidea virens*

**DOI:** 10.3389/fmicb.2021.739453

**Published:** 2021-09-13

**Authors:** Mina Yu, Junjie Yu, Huijuan Cao, Tianqiao Song, Xiayan Pan, Zhongqiang Qi, Yan Du, Rongsheng Zhang, Shiwen Huang, Wende Liu, Yongfeng Liu

**Affiliations:** ^1^Institute of Plant Protection, Jiangsu Academy of Agricultural Science, Nanjing, China; ^2^State Key Laboratory of Rice Biology, China National Rice Research Institute, Hangzhou, China; ^3^State Key Laboratory for Biology of Plant Diseases and Insect Pests, Institute of Plant Protection, Chinese Academy of Agricultural Sciences (CAS), Beijing, China

**Keywords:** *Ustilaginoidea virens*, rice false smut, pathogenicity, growth, SUN protein

## Abstract

*Ustilaginoidea virens*, the causal agent of rice false smut disease, is an important plant pathogen that causes severe quantitative and qualitative losses in rice worldwide. UvSUN1 is the only member of Group-I SUN family proteins in *U. virens*. In this work, the role of UvSUN1 in different aspects of the *U. virens* biology was studied by phenotypic analysis of *Uvsun1* knockout strains. We identified that UvSUN1 was expressed during both conidial germination and the infection of rice. Disruption of the *Uvsun1* gene affected the hyphal growth, conidiation, morphology of hyphae and conidia, adhesion and virulence. We also found that UvSUN1 is involved in the production of toxic compounds, which are able to inhibit elongation of the germinated seeds. Moreover, RNA-seq data showed that knockout of *Uvsun1* resulted in misregulation of a subset of genes involved in signal recognition and transduction system, glycometabolism, cell wall integrity, and secondary metabolism. Collectively, this study reveals that *Uvsun1* is required for growth, cell wall integrity and pathogenicity of *U. virens*, thereby providing new insights into the function of SUN family proteins in the growth and pathogenesis of this pathogen.

## Introduction

*Ustilaginoidea virens* (teleomorph: *Villosiclava virens*) is the causal agent of rice false smut (RFS) disease. which is responsible for significant quantitative and qualitative losses in the majority of rice planting areas worldwide (Sun et al., 2020). The most visible symptom of infected rice panicles is the small whitish smut balls that appear first between the glumes, and then continue to grow and enclose the floral parts (Fu et al., 2013; Tang et al., 2013). At the late phase of infection, balls develop a mass of powdery dark green chlamydospore. Moreover, *U. virens*, especially its false smut balls, can produce a variety of cyclopeptide mycotoxins, which are deleterious to human and animal health and pose a serious threat to food security and sustainable rice production (Zhang et al., 2014; Li et al., 2019).

In recent years, with the release of the *U. virens* genome and the improvement of targeted gene deletion method (Zhang et al., 2014; Liang et al., 2018), the functions of several proteins in *U. virens* have been deeply studied (Fang et al., 2019; Xie et al., 2019; Zhang et al., 2020; Chen et al., 2021). The transcription factors UvPRO1 and UvCom1 play critical roles in both hyphal growth and conidiation, as well as in stress response and pathogenesis (Lv et al., 2016; Chen et al., 2020a). A homeobox transcription factor UvHOX2 governs chlamydospore formation and pathogenicity in *U. virens* (Yu J. et al., 2019). The conserved mitogen-activated protein kinases (MAPKs) partially regulate pathogenicity in multiple phytopathogenic fungi (Sun et al., 2020). In *U. virens*, MAPK proteins UvHog1, UvCDC2, UvSLT2 and UvPmk1 have conserved roles in regulating stress responses, hyphal growth, and secondary metabolism, and the last three proteins were further confirmed to be associated with pathogenicity experimentally (Zheng et al., 2016; Liang et al., 2018; Tang et al., 2019). cAMP signaling pathway-related proteins, cyclase-associated proteins UvCAP1, adenylate cyclase UvAc1 and phosphodiesterase UvPdeH also involved in regulating the intracellular Cyclic adenosine monophosphate (cAMP) level, development, and pathogenicity of *U. virens* (Guo et al., 2019; Cao et al., 2021). The “pears and lemons” protein UvPal1 physically interacted with UvCdc11 to mediate the septin complex to maintain the cellular morphology and virulence of *U. virens* (Chen et al., 2020b). Putative phosphatase UvPsr1 and UvAtg8-mediated autophagy were also required for mycelial growth, conidiation, stress response and pathogenicity (Meng et al., 2020; Xiong et al., 2020). These results provide an important theoretical basis for understanding the molecular mechanisms of *U. virens*. However, despite these significant advances, due to its slow growth rate and the difficulty of pathogenicity detection, understanding of the unique pathogenic mechanism of *U. virens* in rice is still very limited and needs to be further experimentally verified.

The SUN protein family is derived from four homologous genes *SIM1*, *UTH1*, *NCA3* and *SUN4* of *Saccharomyces cerevisiae* (Firon et al., 2007). It is a unique protein family in ascomycetes. The C-terminal of SUN protein has a highly conserved sequence of 258 amino acids (SUN domain, pfam03856), which contains a Cys-X_5_-Cys-X_3_-Cys-X_24_-Cys motif. According to the number of amino acids between the third and fourth cysteines of the conserved motif, SUN protein can be divided into two groups, namely Group-I with 24 amino acids between the two cysteines and Group-II with multiple insertions between these two cysteines (Firon et al., 2007). Up to now, the SUN proteins that have been found and studied are mainly in yeast, involving in nDNA replication, cell septation, cell wall morphogenesis, mitochondrial biogenesis, stress response, aging processes and other physiological activities (Hiller et al., 2007; Ritch et al., 2010; Sorgo et al., 2013). *Candida albicans* sun41p has also been confirmed to be associated with pathogenicity (Hiller et al., 2007; Firon et al., 2007; Sorgo et al., 2013).

In filamentous fungi, to our knowledge, two Group-I SUN family proteins have been experimentally studied. AfSUN1 from *Aspergillus fumigatus*, the causal agent of aspergillosis in humans, was reported to be involved in fungal morphogenesis (Gastebois et al., 2013). Furthermore, Gastebois et al. (2013) studied the biochemical characteristics of *A. fumigatus* Afsun1p and *Candida albicans* Sun41p, showing that they can specifically hydrolyze straight chain β-(1, 3)-glucan, and represents a new glucan hydrolase family (GH132). BcSUN1, which contains a signal peptide for secretion and potentially hyper-*O*-glycosylated regions, is involved in maintaining the structure of the cell wall, the extracellular matrix and the pathogenesis in *Botrytis cinerea*, a necrotrophic plant fungal pathogen (González et al., 2012; Pérez-Hernández et al., 2017). Moreover, functions of the Group-II SUN family proteins in filamentous fungi are different. Deletion of *AfSUN2* in *A. fumigatus* did not result in any phenotypic difference relative to the parental strain (Gastebois et al., 2013). However, in *U. virens*, the Group-II SUN family protein UvSUN2 has been proposed to be involved in growth and response to stress (Yu et al., 2015). Therefore, SUN proteins may play various roles in different fungi.

Here, we identified a Group-I SUN family protein UvSUN1 in *U. virens*, a nonobligate biotrophic fungus. The phenotypic characterization of the *Uvsun1* gene disruption mutant confirmed that UvSUN1 was involved in the regulation of mycelial growth, conidiation, cell wall integrity and pathogenicity in *U. virens*.

## Materials and Methods

### Strains and Growth Conditions

The wild type *U. virens* strain used in this work was P1 (Yu et al., 2015). It was kept as conidial suspensions in 20% glycerol at −80°C for long-term storage, and routinely cultured on YTA (0.1% yeast extract, 0.1% tryptone, and 1% sucrose, supplemented with 1.5% agar). Fungal cultures were routinely incubated at 28°C in the dark. *U. virens* conidia was prepared from cultures in YT (0.1% yeast extract, 0.1% tryptone, and 1% sucrose) in a 28°C shaker (150 rpm) for 7 days. *Oryza sativa* L. spp. *Indica* cultivar LYP9 (highly susceptible) was grown at the experiment station in Nanjing, Jiangsu, China.

### *Uvsun1* Gene Deletion and Complementation

To obtain the *Uvsun1* gene deletion mutants, the gene replacement vector (pMD19-T-*Uvsun1KO*) and the corresponding pCas9-tRp-gRNA vector (pCas9-tRp-gRNA-*Uvsun1*) were co-transformed into protoplasts of wild type strain P1. For generation of the pCas9-tRp-gRNA vectors for deletion of *Uvsun1*, the gRNA spacers were designed with the gRNA designer program for best on-target scores. *Uvsun1* gRNA spacer CR1 was selected by weighing both^1^ on-target scores and potential off-targets. The sense and antisense oligonucleotides synthesis and the pCas9-tRp-gRNA-*Uvsun1* construction were followed as described before (Liang et al., 2018). The *Uvsun1* gene replacement constructs (pMD19-T-*Uvsun1KO*) were generated according to the homologous recombination principle. The 1010 bp upstream and 996 bp downstream flanking sequences of *Uvsun1* were amplified with primer pairs of S1F/S1R and S2F/S2R, respectively, and fused with the 1396 bp hygromycin-resistance cassette (*Hph*) (amplified with primers: HF and HR) from pSK1044 (Yu et al., 2015) by ClonExpress^TM^ MultiS One Step Cloning Kit (Vazyme) to the pMD19-T vector (Takara). Protoplast preparation and recovery of hygromycin-resistant transformants were performed as described previously (Guo et al., 2019; Meng et al., 2020). For complementation, a fragment containing the entire *Uvsun1* gene and its native promoter region (upstream 1.5 kb sequence) were amplified with primer pairs of pKO1-SC1F/pKO1-SC1R. The gene complement vector construction and *Agrobacterium*-mediated transformation protocol were performed as described previously (Yu et al., 2015; Yong et al., 2020). All constructs were confirmed by sequencing. The resulting transformants were confirmed by PCR with primer pairs (SyF/HR and SyR/HF) and sequencing. Mycelia were harvested from 7-day-old cultures grown in YT and used for genomic DNA extractions.

### Sequence Analysis

Hidden Markov models (HMM) profile and Basic Local Alignment Search Tools (BLAST) searches were performed on the *U. virens* protein database as described previously (Yu M. et al., 2019). The information of domain architecture, introns and exons of the *Uvsun1* were obtained from NCBI. Multiple sequence alignments were aligned using DNAMAN. Potential glycosylation sites were predicted by NetOGlyc 4.0 (González et al., 2014). Phylogenetic analysis was based on the neighbor-joining algorithm with MEGA7.

### RNA Isolation and Quantitative Real-Time PCR (qRT-PCR) Analysis

The abundance of *Uvsun1* transcript was estimated using qRT-PCR assays. For the transformants confirmation assay, mycelia were harvested from 5-day-old cultures grown in YT. For the germinated conidia expression assays, to initiate the cultures, conidia were collected from 7-day-old YT cultures, filtered with one-layer Miracloth (EMD Millipore Crop, United States), then collected by centrifugation and diluted with sterile water to a concentration of 2 × 10^6^ conidia/mL. The same amount of conidia were coated onto a sterilized cellophane membrane on a YTA plate. At 28°C, the germ tube produced by conidial germination could be seen at 12–14 h post incubation (hpi) in the dark. Then the hyphae produced branches at about 24 hpi and continued to grow until 72 hpi, when conidia were produced at the tips of the hyphae. These germinated conidia were sampled together with the cellophane at 0, 12, 18, 24, 48, and 72 hpi. For the *in planta* expression studies, the WT strain was sampled *in planta* at 0, 1, 2, 3, 5, 7, and 14 dpi (days post inoculation) as described before (Han et al., 2015).

RNA was extracted from the samples using an RNA isolation kit (BioTeke). One microgram of total RNA was used as template for cDNA synthesis using a Primescript^TM^ RT reagent kit with gDNA Eraser (TaKaRa), according to the manufacturer’s instructions. qRT-PCR reactions were performed in a QuantStudio3 (Thermo Fisher) with the SYBR *Premix Ex Taq*^TM^ II kit (Takara) and the primers listed in Supplementary Table 1. The *β-tubulin* sequence was chosen as the endogenous reference. The relative mRNA amounts were calculated by the −2^△△Ct^ method as described before (Pérez-Hernández et al., 2017). Data from three biological replicates were used to calculate the mean and standard deviation.

### Phenotypic Analysis

We used CM medium to test mycelial growth rate and YT to test conidiation ability of *U. virens* (Yu et al., 2015). The sensitivity of strains to a range of abiotic stress agents were tested by culturing them at 28°C for 12 d on YTA medium supplemented with one of the following chemicals: 0.4 M NaCl, 0.8 M sorbitol, 3 mM H_2_O_2_, 0.03% sodium dodecyl sulfate (SDS) or 400 μg/mL calcofluor white (CFW). The inhibition rates were calculated as described previously (Xie et al., 2019).

The dry weight was calculated after the mycelium was completely dried. The same amount of conidia were inoculated on the sterilized cellophane on YTA medium for 5 days incubation at 28°C in the dark. Then, the mycelia were collected from the cellophane and dried at 80°C to a constant weight.

Toxicity assays were carried out by challenging the ability of seeds (LYP9) to germinate in the presence of YT culture filtrate (Zheng et al., 2016). The uninoculated YT was used as the control. Each germination assay (shoot and root growth) comprised 50 seeds was replicated three times.

To compare the amount of ECM around the hyphae, the fungus was grown for 7 days in 50 mL of YT (inoculated with 1 × 10^6^ conidia/mL). The mycelium was then collected and completely overlaid with several drops of black India ink, covered with a coverslip, and observed under the microscope (Pérez-Hernández et al., 2017).

Film studies were based on the method described by Gravelat et al. (2010). 12-well plates were inoculated with 1 mL per well of YT containing 1 × 10^6^ conidia/mL. After 24 h incubation at 28°C with shaking at 120 rpm, 500 μL fresh YT was added to each well. After a further 24 h incubation, the spent culture medium was removed from each well and the adherent cells were washed three times with PBS. Film density was estimated by staining with 500 μL 0.5% (w/v) crystal violet solution for 5 min. The films were then gently washed with running water and destained by adding 1 mL of 95% ethanol to each well. Absorbance measurements of the destaining solution were made at 520 nm to estimate the density of the film.

For SEM assay, hyphae were grown for 7 days in YT. Then hyphae were collected and fixed with 2.5% glutaraldehyde in 0.1 M PBS at 4°C overnight, sequentially dehydrated in ethanol, and critical-point dried (Gravelat et al., 2010). Samples were then Au-Pd sputter-coated and imaged with a scanning electron microscope (EVO-LS10, Zeiss).

Pathogenicity tests were performed as described by Yu et al. (2015). The strains were propagated on YTA plates for 10 days at 28°C in the dark. Then six 5 mm-diameter mycelia discs were cut from the edge of the colony and inoculated in 50 mL YT with shanking at 28°C for 7 days. Mixtures of mycelia and conidia were harvested and mixed with a blender. Then the conidia concentration of the mixtures was adjusted to 1 × 10^6^/mL with YT. One mL of this inoculum was injected into the swollen sheaths of flag leaves on the main stems one week before rice heading using sterilized syringes. Twenty-one days after inoculation, the number of rice false smut balls per panicle was evaluated. At least 10 panicles were inoculated with each transformant at each time.

All the experiments were performed with three replicates.

### Comparative Transcriptional Analysis

Total RNA of *U. virens* was isolated from the mycelia of the P1 or △*Uvsun1* strains at 5 day post-inoculation in YT using TRIzol Reagent according to the manufacturer’s instructions (Invitrogen). The concentration and purity of RNA were detected by Nanodrop2000. The integrity of RNA was detected by agarose gel electrophoresis, and the RIN value measuring by Agilent2100. RNA-seq libraries were prepared using an Illumina TruSeq RNA Sample Preparation Kit (San Diego, Ca). Double-stranded cDNA was synthesized using a SuperScript double-stranded cDNA synthesis kit (Invitrogen, CA) with random hexamer primers (Illumina). After quantified by TBS380, cDNA library was sequenced using Illumina Novaseq 6000 (2 × 150 bp read length). All the experiments were carried out at the Majorbio company (Shanghai, China).

The raw paired-end reads were trimmed and quality controlled by SeqPrep^2^ and Sickle^3^ with default parameters. The generated clean data were then separately aligned to *U. virens* Uv8b genome (NCBI) with orientation mode using HISAT2^4^ software (Kim et al., 2015). The mapped reads of each sample were assembled by StringTie^5^ in a reference-based approach (Pertea et al., 2015). Differential gene expression between two samples was identified according to the transcripts per million reads (TPM) method. RSEM^6^ was used to quantify gene abundances (Li and Dewey, 2011). Differential expression analysis was performed using the DESeq2 with the criteria of | Log2FC| ≥ 1 and Padjust < 0.05 (Love et al., 2014). In addition, functional-enrichment analysis including GO and KEGG were performed to identify which DEGs were significantly enriched in GO terms and metabolic pathways at Bonferroni-corrected P-value ≤ 0.05 compared with the whole-transcriptome background. GO functional enrichment and KEGG pathway analysis were carried out by Goatools^7^ and KOBAS^8^ (Xie et al., 2011). Three biological replicates were performed for each strain. The raw data of the RNA-seq was deposited in NCBI (accession number: PRJNA746442).

### Statistical Analysis

Statistical analysis for a one-way Analysis of Variance (ANOVA) was carried out with SAS system. Data are shown as mean ± SD of three independent replicates. Asterisks indicate a statistically significant difference with the wild type strain (*p* < 0.05).

## Results

### Identification of the *Uvsun1* Gene in *U. virens*

The HMM profile and a BLAST search against the *U. virens* genome identified two SUN domain-containing proteins UvSUN1 (KDB16044) and UvSUN2 (Yu et al., 2015) (Supplementary Figure 1). Aligning these two sequences with the SUN proteins from *S. cerevisiae*, UvSUN1 showed an overall identity ranging from 41% for NCA3 and SIM1 to 42% for UTH1 and SUN4, that belong to Group-I of the SUN family (Table 1 and Supplementary Figure 2). While UvSUN2 showed 50% amino acid identity with the hypothetical protein YMR244W from *S. cerevisiae*, which classified it as a member of the Group-II of the SUN family, as described pervious (Yu et al., 2015). The full length of the *Uvsun1* gene was 1925 bp, consisting of 197 bp 5′-UTR, 354 bp 3′-UTR, a 68 bp intron and a 1374 bp open reading frame, coding for a protein of 457 amino acids. The SUN domain of UvSUN1 contains the canonical Cys-X_5_-Cys-X_3_-Cys-X_24_-Cys motif, spanned residues 111–414 (Supplementary Figure 2). Furthermore, UvSUN1 was predicted to be highly glycosylated by NetOGlyc 4.0. Similar to as for *A. fumigatus*, and *B. cinerea*, *U. virens* contained only one Group-I SUN protein UvSUN1, showing similarities in SUN domain sequences and gene structure to other Group-I SUN proteins (Supplementary Figure 2).

**TABLE 1:** Amino acid sequence identity and expect value of UvSUN1 and UvSUN2 to *S. cerevisiae* proteins in the SUN family.

Organism	*Saccharomyces cerevisiae*					*Ustilaginoidea virens*	
	SIM1	UTH1	NCA3	SUN4	YMR244W	UvSUN1	UvSUN2
SIM1		65%	66%	79%	32%	41%	33%
UTH1	2.E-93		72%	59%	36%	42%	33%
NCA3	1.E-96	1.E-100		58%	31%	41%	34%
SUN4	5.E-133	7.E-92	1.E-92		34%	42%	30%
YMR244W	1.E-27	6.E-31	3.E-29	5.E-26		30%	50%
UvSUN1	1.E-61	1.E-63	1.E-59	2.E-64	4.E-22		31%
UvSUN2	42.E-18	3.E-27	3.E-36	1.E-15	1.E-89	8.E-22	

*Identity and expect value were computed in NCBI from alignments of the entire sequences. NCBI accession numbers for the SUN proteins in *S. cerevisiae* are as follows: P40472 (SIM1), P36135 (UTH1), P32493 (NCA3), and P53616 (SUN4).*

### The Expression of the *Uvsun1* Gene

**FIGURE 1:**
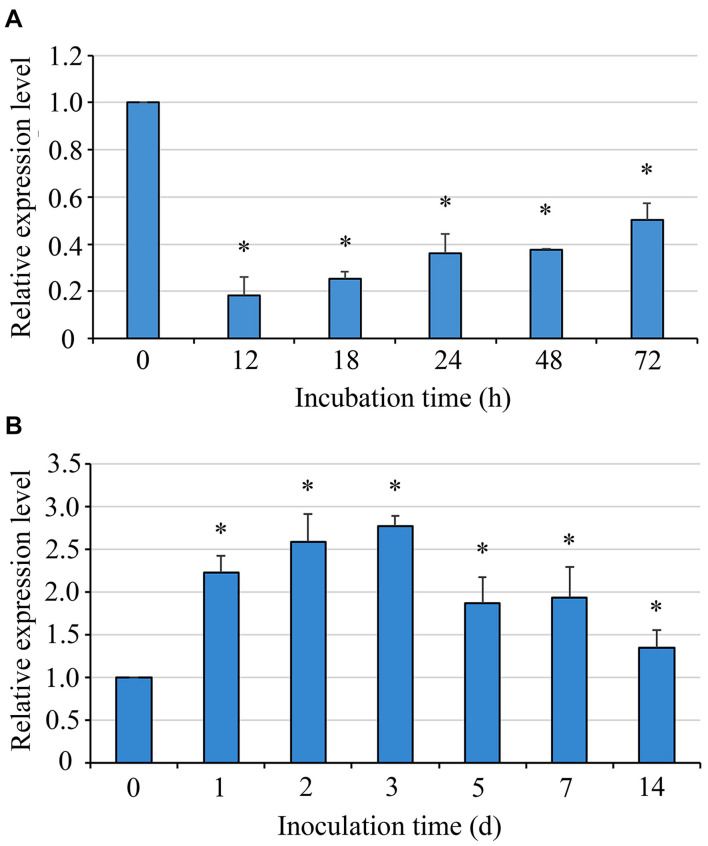
The mRNA level of *Uvsun1* in the wild type strain P1 during conidia germination and rice infection. **(A)** Levels of *Uvsun1* mRNA in the wild type strain P1 during conidia germination. Conidia were incubated on cellophane on YTA medium. Samples were taken at 12, 18, 24, 48, and 72 hpi. Data are relative to the mRNA levels in ungerminated conidia. **(B)** Levels of *Uvsun1* mRNA following the inoculation of rice panicles with P1 at various days after inoculation (dpi). Data are relative to the mRNA levels in mycelium before inoculation. Results are expressed as mean ± SD of three technical replicates. Asterisks indicate significant differences (one-way ANOVA, ^∗^*p* < 0.05).

*Uvsun1* expression was detected by qRT-PCR 12 h after the initiation of incubation and after that during mycelial growth. The results showed that the expression of *Uvsun1* increased gradually during germination and mycelial growth, but its mRNA expression level was lower than that measured in conidia. Furthermore, during infection, qRT-PCR experiments showed that *Uvsun1* transcripts were abundant, increased linearly within 3 days after inoculation and decreased slowly until 14 dpi (Figure 1). These results suggested that *Uvsun1* might have important roles in vegetative growth and pathogenicity of *U. virens*.

### *Uvsun1* Is Involved in Vegetative Growth and Conidiogenesis

To analyze the function of *Uvsun1*, deletion mutants were generated by replacing the gene with a *hygromycin B* resistance cassette in the WT strain P1. PCR amplification from genomic DNA and sequencing analysis confirmed that *Uvsun1* was deleted in three mutants (#33, #44, and #48) (Supplementary Figure 3). Since these *Uvsun1* mutants had similar phenotypes, mutant #44 was selected for additional studies. A complementation assay was carried out with △*Uvsun1-44* to generate the complemented strain C△*Uvsun1*.

**FIGURE 2:**
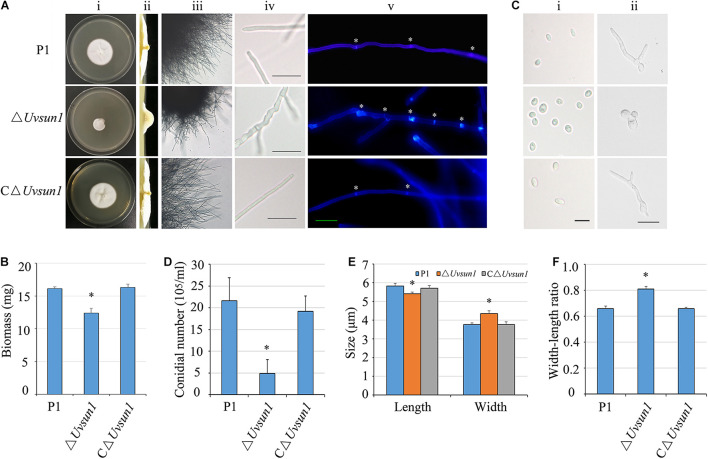
*Uvsun1* affects mycelial growth, conidial morphology and production. **(A)** Colony morphology of wild-type (P1), △*Uvsun1* deletion mutant and the complemented strain C△*Uvsun1* on CM medium after 12 days of incubation at 28°C (i and ii). The mutant strains showed more branched hyphae (iii) and the surface of hyphae expanded irregularly. Scale bars, 20 μm. (iv). (v) Hyphae of wild-type, mutant and complemented strains were stained with Calcofluor white, which detects chitin and cellulose in the cell wall, and fluorescence is shown in blue color. The hyphal septum of the △*Uvsun1* mutant was shorter than that of wild type and complemented strains. Asterisk indicate the hyphal septa. Scale bars, 10 μm. **(B)** Quantified dry weight of wild type, mutant and the complemented strain. **(C)** The conidial morphology and germination of wild-type, mutant and the complemented strain were photographed after culturing on YT for 0 h (i) and 24 h (ii). Scale bars, 10 μm. **(D)** Statistical analysis of conidia production on YT medium after 7 days culture. Data are shown as mean ± SD from three independent replicates. Asterisks indicate significant differences (one-way ANOVA, ^∗^*p <* 0.05). **(E)** and **(F)** The width and length of conidia was statistically analyzed, and error bars represent SD. The asterisks indicate significant differences (one-way ANOVA, ^∗^*p* < 0.05).

The growth rate of deletion mutants △*Uvsun1* was significantly reduced compared to that of P1 and the complemented strain C△*Uvsun1* (Figure 2A). Furthermore, the colony of the mutant strains was more compact, smooth and hyphae branched than P1. The dry weight of hyphae was also measured to evaluate the growth. Expectantly, the mutants showed lower dry weights than that of P1 (Figure 2B). An alteration of the mycelial morphology in the mycelium of the △*Uvsun1* was in agreement with its growth defects. The hyphae of the △*UvSun1* mutants showed short intercalary cells with closely arranged septa and swollen appearance (Figure 2A).

The conidial production of △*Uvsun1* was reduced. Specifically, the P1 produced 21.6 ± 6.3 × 10^6^ conidia/mL, while the mutant produced 4.9 ± 3.2 × 10^6^ conidia/mL (Figure 2D). Furthermore, the conidia produced by △*Uvsun1* mutants showed a more globose structure (Figures 2C,E,F). The germination rate of the △*Uvsun1* mutants showed no difference with P1, but its germinated conidia appeared irregular. That is, only 25.3 ± 5.4% of germinated conidia produced normal filamentous hyphae, while 70.6 ± 3.6% of them showed budding-yeast structures (Figure 2C). These results indicated that *Uvsun1* plays important roles in fungal growth and conidiogenesis in *U. virens*.

### *Uvsun1* Is Involved in Response to Different Abiotic Stresses

**FIGURE 3:**
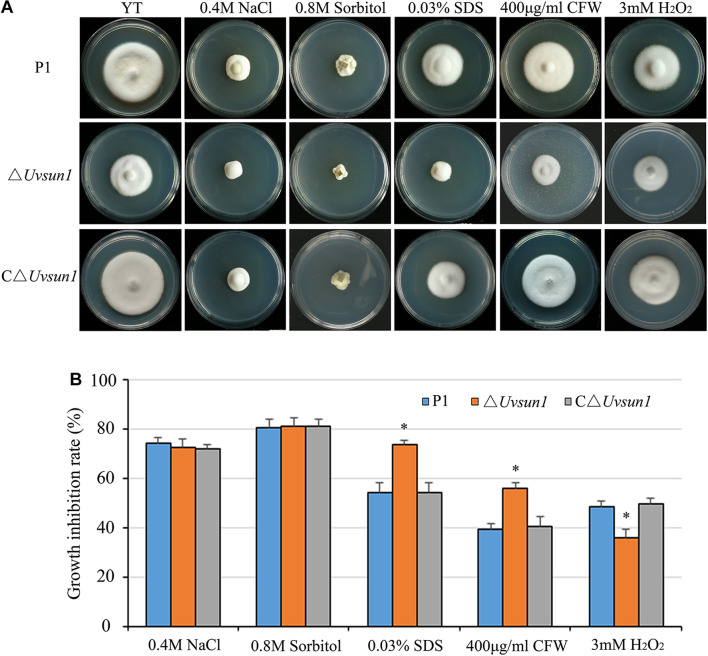
*Uvsun1* is involved in regulating pathogen stress responses. **(A)** Mycelial radial growth of the indicated wild-type P1, △*Uvsun1* and the complemented C△*Uvsun1* strain on YT medium supplemented with salt stress agent (0.4 M NaCl), osmotic stress agent (0.8 M Sorbitol), oxidative-stress agent (3 mM H_2_O_2_), and cell wall disturbing agents Calcofluor white (CFW, 400 mg/mL) and sodium dodecyl sulfate (0.03% SDS). Photographs were taken after 12 days of incubation at 28°C. **(B)** Statistical analysis of the indicated strains growth inhibition rate under different stress conditions. Colony diameters of the indicated strains were measured. Data are shown as mean ± SD of three independent replicates. Asterisks indicate significant differences (one-way ANOVA, ^∗^*p* < 0.05).

The △*Uvsun1* mutants showed no difference with the wild type in the growth rate in YTA media containing 0.4 M NaCl or 0.8 M Sorbitol. Nevertheless, the addition of SDS or Calcofluor white (CFW), which are known to affect the integrity of the cell wall, did cause a significant reduction in the growth rate of the △*Uvsun1* mutants compared to that of P1 and C△*Uvsun1* (Figure 3). This result suggested that UvSUN1 might have a role in the biogenesis or stability of the cell wall. When the strains were cultured on YTA medium containing 3 mM H_2_O_2_, the growth of the △*Uvsun1* mutants was moderately slowed, while those of both the WT and the C△*Uvsun1* strains showed significant reduction (Figure 3). Taken together, these results indicated that *Uvsun1* is required for regulating the *U. virens* responses to cell wall integrity, as well as oxidation stress.

### *Uvsun1* Is Involved in Cell Surface Alterations

**FIGURE 4:**
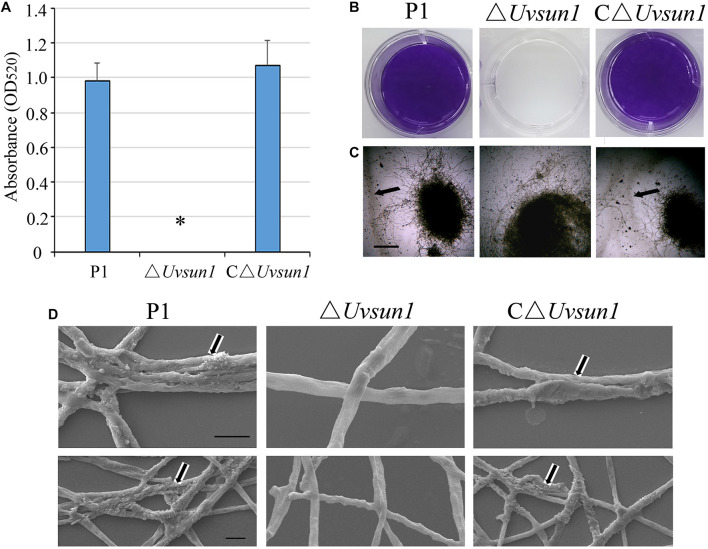
Lack of *Uvsun1* induces cell surface alterations. **(A)** The density of films was estimated by the absorbance measurements of the destaining crystal violet solution at 520 nm. **(B)** Formation of films by hyphae of the indicated strains after 24 h growth on polystyrene plates. After washing, hyphae were stained with crystal violet for visualization. **(C)** ECM displayed by the Δ*Uvsun1* mutant, as compared with the wild type P1 and complemented strain C△*Uvsun1*, detected by India ink staining of conidia germinated in YT medium for 7 days (Scale bars, 200 μm). **(D)** Scanning electron micrographs of hyphae of the indicated strains for 7 days. Magnification was 20,000× (up, Scale bars, 2 μm) and 10,000× (down, Scale bars, 10 μm). All assays were performed on at least three independent occasions.

When the wide-type P1 and the △*Uvsun1* mutants were grown in YT for 7 days, the culture medium of △*Uvsun1* was less viscous, raising the possibility of its involvement in the alteration of extracellular matrix (ECM). To examine this hypothesis, the strains were grown for 7 days in YT and the ECM was negatively stained with India ink. ECM was observed as a clear halo surrounding the mycelium in the wild type P1, while the halo was difficult to find in the △*Uvsun1* mutants (Figure 4C). One of the functions proposed for the ECM is in cell attachment (Pérez-Hernández et al., 2017). The influence of the deletion of *Uvsun1* on the attachment was assessed by determining the density of adherent films, produced by the △*Uvsun1* mutants on plastic, as compared to the wild type. We found that the △*Uvsun1* mutants were markedly impaired in the formation of adherent films (Figures 4A,B). Scanning electron microscopy of △*Uvsun1* mutants showed a complete loss of surface coat and intercellular matrix of hyphae (Figure 4D). Collectively, these results suggested that UvSUN1 was responsible for the ECM and the adherence of *U. virens* to plastic.

### *Uvsun1* Is Involved in Pathogenicity on Host Plants

**FIGURE 5:**
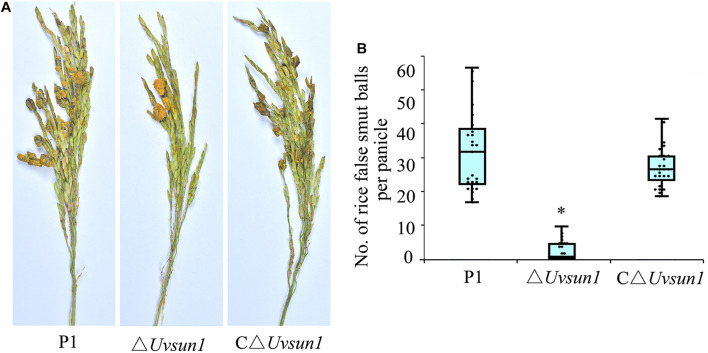
*Uvsun1* is required for *Ustilaginoidea virens* full pathogenicity in rice. **(A)** Rice spikelets were infected with an inoculum of P1, △*Uvsun1* and C△*Uvsun1* strains. Pictures were taken 21 days post-inoculation. **(B)** Quantification of false smut balls per infected panicle. The data were collected from three independent experiments, with a total of at least 25 panicles per line. Asterisks indicate significant differences (one-way ANOVA, ^∗^*p* < 0.05).

To study the effect of *Uvsun1* on fungal virulence, the P1, △*Uvsun1* and C△*Uvsun1* strains were inoculated into panicles of the susceptible rice cultivar LYP9. At 21 days post-inoculation (dpi), the false smut balls produced on rice spikelets inoculated with △*Uvsun1* strains was significantly fewer than those infected by P1 and C△*Uvsun1* strains (Figure 5). These results suggested that *Uvsun1* was required for the pathogenicity of *U. virens.*

### Culture Filtrate of Δ*Uvsun1* Has Increased Phytotoxicity

**FIGURE 6:**
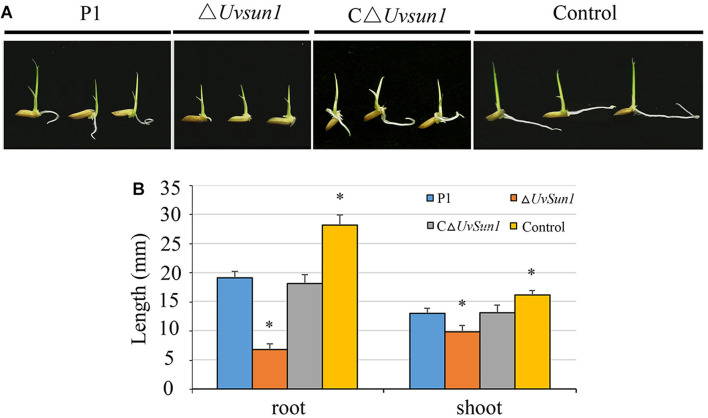
Culture filtrates of Δ*Uvsun1* mutants showed increased inhibition of rice seed germination. **(A)** Grains were germinated in the presence of the filtrate of P1, Δ*Uvsun1*, C△*Uvsun1* or uninoculated YT after 5 days of culture. **(B)** Quantification of root and shoot lengths of the seedling. Data represent means ± SD from three independent experiments. Asterisks indicate significant differences (one-way ANOVA, ^∗^*p* < 0.05).

To investigate whether *Uvsun1* affect the production of phytotoxic compounds, we collected YT culture filtrates from P1, △*Uvsun1*, and C△*Uvsun1* after 5 days of culturing, as well as uninoculated YT to use for rice seed germination assays. Compared with the uninoculated YT, the growth of rice roots and shoots were inhibited by P1 and C△*Uvsun1* culture filtrates after 5 days. However, the lengths of the roots and shoots treated with culture filtrates of Δ*Uvsun1* were significantly shorter than the P1 and the complemented strains (Figure 6). These results showed that more toxicity compounds to rice seed were produced by the Δ*Uvsun1* mutants, suggesting that *Uvsun1* is involved in producing toxic compounds.

### *Uvsun1* Deletion Affects the Transcription of a Subset of Genes

To understand a comprehensive perspective on the function of UvSUN1, we used Illumina sequencing to analyze transcriptome dynamics and differentially expressed genes (DGEs) of P1 and △*Uvsun1* strains. Samples for RNA-seq were extracted from mycelia of the Δ*Uvsun1* mutants and P1 cultured in YT for 7 days. There was high Pearson correlation among duplicates. Analysis of the DGEs, revealed that the deletion of *Uvsun1* affected the transcription of a subset of genes (Table 2).

**TABLE 2:** A list of genes that may be affected by UvSUN1.

Gene	Biological function	Regulate^a^	Log2FC (*Uvsun1*/P1)	e-value
**Genes involved in oxidation-reduction process**				
UV8b_1064	FAD dependent oxidoreductase	down	–1.86065	0.00232
UV8b_1132	putative fatty acid oxygenase	down	–3.19406	4.84E-20
UV8b_3597	oxidoreductase	down	–8.31345	0.00077
UV8b_3614	delta-9 fatty acid desaturase	down	–1.24256	0.00177
UV8b_3300	NAD binding Rossmann fold oxidoreductase	down	–1.21374	0.00051
UV8b_2282	cytochrome P450 52A11	up	7.933256	0.001761
UV8b_4642	flavin-containing monooxygenase	down	–1.40193	7.31E-05
UV8b_4655	NAD(P)-binding domain protein	down	–1.48665	0.001821
UV8b_4656	cytochrome P450	down	–1.04539	0.000811
UV8b_894	flavin-nucleotide-binding protein	down	–2.66365	0.000324
UV8b_7793	flavin-binding monooxygenase	down	–1.96231	0.000305
UV8b_648	inositolphosphorylceramide-B C-26 hydroxylase	down	–1.63302	3.53E-05
UV8b_7046	malate dehydrogenase	down	–2.72295	0.000165
UV8b_7242	Quinate dehydrogenase	down	–2.8423	3.02E-05
UV8b_6593	aldehyde dehydrogenase	down	–1.65112	0.000485
UV8b_6662	zinc-binding dehydrogenase	down	–10.069	1.06E-12
UV8b_683	putative NADPH-dependent alpha-keto amide reductase	down	–1.2823	0.00064
UV8b_6684	salicylate hydroxylase	down	–1.96667	0.000205
**Genes involved in signal transduction**				
UV8b_1542	Protein kinase domain containing protein	up	8.074797	0.001367
UV8b_1384	RING-5 like protein	up	8.832095	4.01E-11
UV8b_3406	serine/threonine-protein kinase SRPK2	up	1.139891	0.000773
UV8b_2973	histone deacetylase phd1	up	12.96718	2.87E-27
UV8b_532	Zn 2Cys6 transcription factor	down	–1.07624	6.62E-06
UV8b_5187	C6 transcription factor	up	2.069936	1.66E-05
UV8b_5199	Ubiquitin-conjugating enzyme	up	1.670267	1.42E-06
UV8b_5734	putative bZip transcription factor	up	6.559539	6.6E-08
UV8b_5932	C2H2 type zinc finger containing protein	up	1.087063	5.33E-07
UV8b_6148	APSES transcription factor	up	1.762713	0.002438
UV8b_6245	putative vacuolar calcium ion transporter	down	–1.65361	0.001708
UV8b_6210	OefC	up	1.575336	2.96E-06
UV8b_6270	putative protein kinase activator (Mob2)	down	–1.47049	6.05E-06
UV8b_6325	Vivid PAS protein VVD	down	–1.41823	5.06E-05
UV8b_780	Leucine Rich Repeat domain protein	up	1.243039	3.3E-05
**Genes involved in cell membrane and cell wall integrity**				
UV8b_6518	integral membrane protein	up	1.104056	0.002281
UV8b_3446	wall-associated proteinase precursor	down	–3.65466	0.002582
UV8b_2799	cell wall surface anchor signal protein	down	–1.13182	2.12E-06
UV8b_4227	putative glycosyl transferase	down	–3.50707	0.000281
UV8b_4102	integral membrane protein	down	–1.1783	2.44E-07
UV8b_4101	cell surface protein	down	–1.39795	0.002038
UV8b_2134	spc97/spc98 family protein	down	–8.50981	0.000702
UV8b_2135	DASH complex subunit ASK1	up	1.186366	0.00027
UV8b_4172	PH-response regulator	down	–2.13291	3.4E-05
UV8b_4210	putative integral membrane protein, Mpv17/PMP22 family	down	–10.9174	1.95E-18
UV8b_4903	putative cell morphogenesis protein Sog2	up	9.168143	1.09E-10
UV8b_4961	putative sodium/phosphate symporter	down	–4.03067	0.000165
UV8b_968	membrane zinc transporter	down	–1.76431	0.00105
UV8b_53	oligopeptide transporter OPT-like protein	down	–4.42234	9.82E-08
UV8b_5292	kinesin related protein 1	up	1.134424	0.000602
UV8b_57	integral membrane protein	down	–1.51656	0.00051
UV8b_7874	putative pirin domain protein	down	–1.42057	5.92E-05
UV8b_8182	GPI anchored cell wall protein	down	–2.01147	0.002074
UV8b_7624	glucose transporter	up	1.384155	1.23E-08
UV8b_7554	putative MFS multidrug transporter	down	–7.86254	0.001973
UV8b_7243	Quinate permease	down	–1.22993	2.11E-07
UV8b_7128	lipase thioesterase family protein	down	–1.51516	0.003283
UV8b_7166	cation efflux family protein family	down	–1.73726	6.16E-08
UV8b_6939	glucose transporter-like protein	down	–1.51809	3.61E-07
UV8b_6993	putative MFS monosaccharide transporter	down	–3.22514	0.002456
UV8b_6806	GPI anchored serine-threonine rich protein	down	–1.97865	0.000102
UV8b_6673	cell wall glycoprotein	down	–3.37874	9.81E-11
UV8b_1099	oligosaccharide transporter, MRT family	down	–1.37709	3.29E-05
UV8b_1668	sugar transporter family protein	down	–1.35407	0.003107
UV8b_1851	MFS multidrug transporter	down	–1.21728	0.002546
UV8b_2742	MFS transporter	down	–3.24606	0.000215
UV8b_25	MFS transporter	down	–1.01519	1.66E-05
UV8b_23	lipid phosphate phosphatase 2	down	–1.0542	7.46E-06
UV8b_2207	putative MFS transporter	down	–1.71115	0.001812
UV8b_3452	mitochondrial phosphate carrier protein	up	2.551245	0.000204
UV8b_3642	N-acetylglucosaminidase	down	–1.2717	2.2E-05
UV8b_3909	invertase precursor	down	–1.20494	5.66E-07
UV8b_2230	ankyrin repeat protein	up	6.191546	0.001113
UV8b_3539	GPR/FUN34 family protein	down	–7.85537	5.61E-05
**Genes involved in glycometabolism**				
UV8b_1061	1,2-a-D-mannosidase	down	–2.39725	0.000199
UV8b_2059	glycosyl hydrolase	up	2.144112	0.002281
UV8b_2474	endopolygalacturonase	down	–3.24606	0.000215
UV8b_3637	class v chitinase	down	–2.98957	0.00237
UV8b_5043	putative polysaccharide synthase Cps1	up	6.29809	2.4E-07
UV8b_7342	glucan endo-1,3-beta-glucosidase	down	–1.57193	0.001121
UV8b_5560	YjeF domain containing protein	up	6.663679	0.000463
UV8b_2578	glycosyltransferase family 90 protein	down	–1.21372	1.35E-05
UV8b_2938	neutral trehalase	up	1.60162	7.87E-05
**Genes involved in secondary metabolism**				
UV8b_8243	p450 monooxygenase	down	–4.99668	6.51E-05
UV8b_7112	laccase-like protein	down	–4.39969	8.36E-07
UV8b_2091	laccase	down	–1.31365	0.00251

*^a^Differential gene expression analysis was based on FPKM, under the criteria of the absolute Log2 Fold change ≥ 1 and Padjust < 0.05.*

**FIGURE 7:**
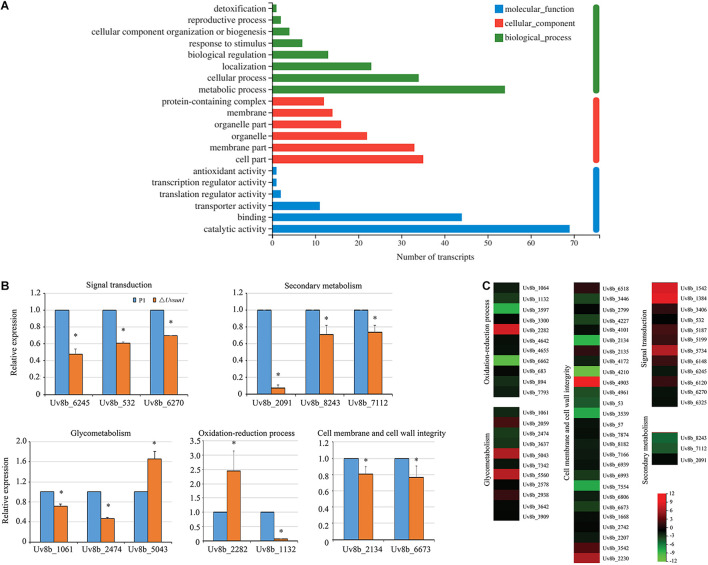
*Uvsun1* regulates the expression of a subset of genes. **(A)** Gene ontology (GO) analysis of significantly differentially expressed genes in △*Uvsun1* mutant compared with WT. The *X*-axis indicates the number of transcripts for each GO item on the *Y*-axis. **(B)** qRT-PCR analysis of *Uvsun1* affected genes. Relative expression levels were normalized with *β-tubulin* as the internal standard. The presented as means ± SD from three biological replicates. Asterisks indicate significant differences (one-way ANOVA, ^∗^*p* < 0.05). **(C)** Differentially expressed genes in the △*Uvsun1* mutants might indicate involvement in pathogenesis and development. Regulated genes include genes involved in signal transduction, secondary metabolism, glycometabolism, oxidation-reduction process and cell membrane and cell wall integrity. The biological functions of the genes are shown in Table 2.

In a total of 8426 genes identified in *U. virens* previously (Zhang et al., 2014), compared with P1, 83 genes showed increased expression and 195 genes showed decreased expression in the Δ*Uvsun1* mutant (Table 2). Gene Ontology (GO) analysis categories indicated that all significantly DEGs were involved in three major functional groups: molecular function, biological process and cellular component (Figure 7A). In the molecular function group, the top three subgroups of DGEs were “metabolic process,” “cellular process,” and “localization.” In the biological process group, the top three subgroups of DGEs were “catalytic activity,” “binding,” and “transporter activity.” In the cellular component group, the top three subgroups of DGEs were “cell part,” “membrane part,” and “organelle.” Enrichment analysis of GO categories indicated that significantly DEGs were enriched mainly in oxidation-reduction process, iron transports and microtubule cytoskeleton (Supplementary Figure 4). Enrichment analysis of Kyoto Encyclopedia of Genes and Genomes (KEGG) categories indicated that significant DEGs were enriched mainly in biosynthesis of unsaturated fatty acids, some amino acid metabolism and glycometabolism pathways (Supplementary Figure 4).

We further analyzed the 278 DEGs and found a subset of genes involved in different processes (Table 2 and Figure 7C). Fifteen DEGs were regarded as components in signal recognition and transduction system, which plays an important role in cell growth, metabolism and response to external environment. qRT-PCR results confirmed the decreased expression of genes encoding putative protein kinase activator Mob2 (Uv8b_6270), putative vacuolar calcium ion transporter (Uv8b_6245) and Zn2Cys6 transcription factor (Uv8b_532) in Δ*Uvsun1* mutants (Figure 7B). Several genes involved in the osmotic response and integrity of the plasma membrane or cell wall were found, including seven upregulated and 29 downregulated genes. Many of them are involved in the integral component of the membrane and cytoplasmic microtubule organization. qRT-PCR results confirmed decreased expression of gamma-tubulin components spc97/spc98 family protein (Uv8b_2134) and cell wall glycoprotein (Uv8b_6673). Consistent with the tolerance to oxygen stress, a series of redox related genes were affected in the Δ*Uvsun1*, including one cytochrome P450 (Uv8b_2282) gene which was increased in expression and 17 genes reduced in expression such as putative fatty acid oxygenase (Uv8b_1132) (Figure 7B). Taken together, these results suggested that *Uvsun1* altered the expression of a subset of genes to affect the growth of *U. virens*.

Previously, a study showed that the fungal SUN proteins represent a new family of glucan hydrolases GH132 (Gastebois et al., 2013). Expectedly, RNA-seq data showed that eleven glycometabolism-related genes were misregulated in △*Uvsun1* mutants (Table 2 and Supplementary Figure 5). Several glycosyl hydrolases like 1,2-α-D-mannosidase (Uv8b_1061, belong to GH47) and endopolygalacturonase (Uv8b_2474) were downregulated in the mutants, while the expression was increased for putative polysaccharide synthase Cps1 (Uv8b_5043) (Figure 7B). The results suggest that *Uvsun1* is related to the glycometabolism in *U. virens*.

## Discussion

SUN family proteins are evolutionarily conserved protein, which have been studied in a small number of ascomycetes. In yeast, they were involved in many physiological activities such as cell partition, cell wall morphogenesis, mitochondrial biogenesis and autophagy, stress response and aging processes (Hiller et al., 2007; Kuzentsov et al., 2013; Pérez-Hernández et al., 2017). In filamentous fungi, AfSUN1 in *A. fumigatus* was involved in hyphal growth, conidiation and cell wall biogenesis (Gastebois et al., 2013). Deletion of *Bcsun1* in *B. cinerea* affected the growth and development, cell wall integrity and pathogenicity of *B. cinerea* (Pérez-Hernández et al., 2017). In this study, we cloned *Uvsun1*, the only member of Group-I of the SUN family in *U. virens*. UvSUN1 was also predicted to have a signal peptide and as hyper-*O*-glycosylated, similar to BcSUN1 in structure (González et al., 2012, 2014; Pérez-Hernández et al., 2017). Deletion of *Uvsun1* decrease the hyphal growth, conidiation and cell wall integrity, but increase the oxidative stress tolerance, and almost completely abolished the fungal pathogenicity. Our results demonstrate that UvSUN1 plays an important role in *U. virens* and shares the conserved function of SUN proteins among different fungal species.

Deletion of *Uvsun1* affected the morphology of hyphae and conidia. Microscopic observation of hyphae showed that the ramifications of the hyphae increased, and the surface of hyphae expanded irregularly with shorter interval of hyphae in the △*Uvsun1* mutants. In addition, the conidia were round and bulky, and the morphology of most conidia was abnormal after germination in △*Uvsun1*. These colony and conidia morphology were similar to that of the △*Afsun1* mutants in *A. fumigatus.* AfSUN1 was confirmed as a glucoside hydrolase GH132 protein with exo-(1,3)-glucanase and minor transferase activities, which act to provide building blocks to other enzymes that are necessary for cell wall biogenesis and/or counteracting the activity of cell wall-degrading enzymes (Gastebois et al., 2013). The cell wall of fungi is predominantly composed of fibrillar and branched β-(1,3)-glucan linked to chitin. β-(1,3)-glucanases are essential for proper conidial cell wall morphogenesis and assembly, and segregation of conidia during conidiation and cell wall in *A. fumigatus* (Mouyna et al., 2016; Millet et al., 2019). Thus, the alterations in the Δ*Uvsun1* mutants regarding morphology of hyphae and conidia, may be consequences of an altered cell wall. Moreover, using cell wall and membrane perturbing agents caused a reduction in the growth rates of △*Uvsun1*. These confirmed that *Uvsun1* affected the cell wall and membrane integrity of *U. virens*. It is consistent with results from other fungi. In yeast, four *S. cerevisiae* SUN proteins were associated with remodeling of the cell wall (Ritch et al., 2010; Kuzentsov et al., 2013). PSU1 from *S. pombe* was also involved in regulating the cell separation. BcSUN1 from *B. cinerea* was reported to be involved in fungal morphogenesis and cell wall remodeling as well (Pérez-Hernández et al., 2017).

Conidia of *U.virens* can be produced by ascospores and chlamydospores, and colonize epiphytically on the leaves and leaf sheaths of rice under suitable temperature and humidity (Tang et al., 2013; Sun et al., 2020). Adhesion to epithelial cells is important for this long period of epiphytic growth and infection. In this study, we found that the deletion of *Uvsun1* affect the production of ECM and the adhesion of *U. virens* to abiotic surfaces. ECM plays an important role in maintaining the morphology and structure of cells, and has an important influence on cell to cell aggregation and adhesion to the surface of objects in many plant pathogens, such as *Magnaporthe oryzae*, *Colletotrichum spp.*, *B. cinerea*, and *Ustilago violace* (Geoghegan et al., 2017). These results are consistent with earlier studies in yeast (Norice et al., 2007). In filamentous fungi, BcSUN1 was also involved in the altered cell wall and ECM, which may cause the alterations of colony morphology (Pérez-Hernández et al., 2017).

The expression pattern of *Uvsun1* in the infection stage showed that *Uvsun1* play a role in the early stage of infection. This result was not consistent with the role of BcSUN1 in the fungus-plant interaction, as the expression levels of BcSUN1 increased up to the late stages of infection when the lesions become necrotic (Pérez-Hernández et al., 2017). These suggested different roles of SUN1 protein in two pathogens with different infection modes. Furthermore, the △*Uvsun1* mutants showed a decrease in the number of false smut balls produced on rice spikelets than P1 and C△*Uvsun1* strains. Altogether, these results suggested that *Uvsun1* is involved in the pathogenic process of *U. virens*, and its main role occurs in the early stage of infection. The △*Uvsun1* mutants were affected in the vegetative growth and the production of conidia and adhesion, which may in turn affect the dispersal of the pathogen.

Glycoside hydrolases hydrolyze the glycosidic bonds between two or more carbohydrates or between carbohydrates and non-carbohydrates (such as protein, lipid) to form monosaccharides, oligosaccharides or glycoconjugates (Breeanna et al., 2007). Pathogens produce numerous glycoside hydrolases, to continuously reshape the cell wall structure of fungal pathogens and to overcome the plant cell wall, during pathogen infection processes (Kubicek et al., 2014; Martins et al., 2019). In △*Uvsun1* mutants, we found a set of misregulated glycoside hydrolases genes. For example, the expression of Uv8b_2474, annotated as a cell wall glycoprotein endopolygalacturonase, was significantly lower in △*Uvsun1*. Endopolygalacturonase activity is vital to fungi and is associated with conidial separation, increased chitin in conidial cell walls, germination, appressorium formation, as well as osmotic and cell wall stress and virulence (Plaza et al., 2020). Endopolygalacturonase-encoding genes *Bcpg1* and *Bcpg2* are required for full virulence of *B. cinerea*. The inactivation of these two genes by gene knockout produced mutants with a significant decrease in virulence (Cettul et al., 2008). Endopolygalacturonase MfPG1 affecting fungal virulence of *Monilinia fructicola* (Chou et al., 2015). In *Lasiodiplodia theobromae*, LtEPG1 functions as an endopolygalacturonase and also serves as an elicitor to manipulate the host immune system and promote its own successful infection and symptom development during infection (Kandawatte et al., 2020). Moreover, the expression of six genes encoding Major Facilitator Superfamily (MFS) transporters in △*Uvsun1* mutants was affected. The MFS is a characterized superfamily of transmembrane secondary transport proteins, essential for uptaking of nutrients and substances necessary for biofilm formation, as well as communication between cells and environment (Li et al., 2017; Wang et al., 2020). *Alternaria alternata* lacking *AaMFS54* produced fewer conidia and increased sensitivity to many potent oxidants (Lin et al., 2018). In *Penicillium digitatum*, Pdmfs2 is required for prochloraz resistance, conidiation and full virulence (Wu et al., 2016).Thus, *Uvsun1* affect the expression of several pathogenicity-related genes, which may also contribute to their altered virulence. Further analysis of the DGEs will contribute to better understanding of the *U. virens* cell wall remodeling and pathogenicity phenotypes found in the *Uvsun1* mutants.

## Data Availability Statement

The datasets presented in this study can be found in online repositories. The names of the repository/repositories and accession number(s) can be found below: https://www.ncbi.nlm.nih.gov/sra/PRJNA746442.

## Author Contributions

SH, WL, and YL conceived and designed the experiments. All authors participated in the editing and approved its final version, design of the experiments, and the analysis/evaluation of the results.

## Conflict of Interest

The authors declare that the research was conducted in the absence of any commercial or financial relationships that could be construed as a potential conflict of interest.

## Publisher’s Note

All claims expressed in this article are solely those of the authors and do not necessarily represent those of their affiliated organizations, or those of the publisher, the editors and the reviewers. Any product that may be evaluated in this article, or claim that may be made by its manufacturer, is not guaranteed or endorsed by the publisher.

## Footnotes

1
https://portals.broadinstitute.org/gppx/crispick/public

2
https://github.com/jstjohn/SeqPrep

3
https://github.com/najoshi/sickle

4
http://ccb.jhu.edu/software/hisat2/index.shtml

5
https://ccb.jhu.edu/software/stringtie/index.shtml?t=example

6
http://deweylab.biostat.wisc.edu/rsem/

7
https://github.com/tanghaibao/Goatools

8
http://kobas.cbi.pku.edu.cn/home.do

## References

[B1] Breeanna R. U. Alan B. B. Elena del C. Carmen C. Takahisa H. Bernard H. (2007). Structural organization and a standardized nomenclature for plant endo-1,4–glucanases (cellulases) of glycosyl hydrolase family 9. *Plant Physiol.* 144 1693–1696. 10.1104/pp.107.102574 17687051 PMC1949884

[B2] Cao H. J. Zhang J. J. Yong M. L. Yu M. N. Liu Y. F. (2021). The cyclase-associated protein uvcap1 is required for mycelial growth and pathogenicity in the rice false smut fungus. *Phytopathol. Res.* 3:5. 10.1186/s42483-021-00083-0

[B3] Cettul E. Rekab D. Locci R. Firrao G. (2008). Evolutionary analysis of endopolygalacturonase-encoding genes of Botrytis cinerea. *Mol. Plant Pathol.* 9 675–685. 10.1111/j.1364-3703.2008.00492.x 19018996 PMC6640430

[B4] Chen X. Hai D. Tang J. Liu H. Huang J. Luo C. (2020a). UvCom1 is an important regulator required for development and infection in the rice false smut fungus *Ustilaginoidea virens*. *Phytopathology* 110 483–493. 10.1094/PHYTO-05-19-0179-R 31638486

[B5] Chen X. Li X. Li P. Chen X. Liu H. Huang J. (2021). Comprehensive identification of lysine 2-hydroxyisobutyrylated proteins in *Ustilaginoidea virens* reveals the involvement of lysine 2-hydroxyisobutyrylation in fungal virulence. *J. Integr. Plant Biol.* 63 409–425. 10.1111/jipb.13066 33427395

[B6] Chen X. Tang J. Pei Z. Liu H. Huang J. Luo C. (2020b). The ‘pears and lemons’ protein UvPal1 regulates development and virulence of *Ustilaginoidea virens*. *Environ. Microbiol.* 22 5414–5432. 10.1111/1462-2920.15284 33073491

[B7] Chou C. M. Yu F. Y. Yu P. L. Ho J. F. Bostock R. M. Chung K. R. (2015). Expression of five endopolygalacturonase genes and demonstration that MfPG1 overexpression diminishes virulence in the brown rot pathogen Monilinia fructicola. *PLoS One* 10:e0132012. 10.1371/journal.pone.0132012 26120831 PMC4488289

[B8] Fang A. Gao H. Zhang N. Zheng X. Qiu S. Li Y. (2019). A novel effector gene SCRE2 contributes to full virulence of *Ustilaginoidea virens* to rice. *Front. Microbiol.* 10:845. 10.3389/fmicb.2019.00845 31105658 PMC6492501

[B9] Firon A. Aubert S. Iraqui I. Guadagnini S. Goyard S. Prévost M. C. (2007). The SUN41 and SUN42 genes are essential for cell separation in Candida albicans. *Mol. Microbiol.* 66 1256–1275. 10.1111/j.1365-2958.2007.06011.x 18001349

[B10] Fu R. Yin C. Liu Y. Ding L. Zhu J. Zheng A. (2013). The influence of nutrient and environmental factors on mycelium growth and conidium of false smut *Villosiclava virens*. *Afri. J. Microbiol. Res.* 7 825–833. 10.5897/AJMR2012.2293

[B11] Gastebois A. Aimanianda V. Bachellier-Bassi S. Nesseir A. Firon A. Beauvais A. (2013). SUN proteins belong to a novel family of β-(1,3)-glucan-modifying enzymes involved in fungal morphogenesis. *J. Biol. Chem.* 288 13387–13396. 10.1074/jbc.M112.440172 23508952 PMC3650377

[B12] Geoghegan I. Steinberg G. Gurr S. (2017). The role of the fungal cell wall in the infection of plants. *Trends Microbiol.* 25 957–967. 10.1016/j.tim.2017.05.015 28641930

[B13] González M. Brito N. González C. (2012). High abundance of serine/threonine-rich regions predicted to be hyper-O-glycosylated in the extracellular proteins coded by eight fungal genomes. *BMC Microbiol.* 12:213. 10.1186/1471-2180-12-213 22994653 PMC3579731

[B14] González M. Brito N. González C. (2014). Identification of glycoproteins secreted by wild-type Botrytis cinerea and by protein-O-mannosyltransferase mutants. *BMC Microbiol.* 14:254. 10.1186/s12866-014-0254-y 25305780 PMC4197228

[B15] Gravelat F. N. Ejzykowicz D. E. Chiang L. Y. Chabot J. C. Urb M. Macdonald K. D. (2010). *Aspergillus fumigatus* MedA governs adherence, host cell interactions and virulence. *Cell. Microbiol.* 12 473–488. 10.1111/j.1462-5822.2009.01408.x 19889083 PMC3370655

[B16] Guo W. Gao Y. Yu Z. Xiao Y. Zhang Z. Zhang H. (2019). The adenylate cyclase UvAc1 and phosphodiesterase UvPdeH control the intracellular cAMP level, development, and pathogenicity of the rice false smut fungus *Ustilaginoidea virens*. *Fungal. Genet. Biol.* 129 65–73. 10.1016/j.fgb.2019.04.017 31063805

[B17] Han Y. Zhang K. Yang J. Zhang N. Fang F. Zhang Y. (2015). Differential expression profiling of the early response to *Ustilaginoidea* virens between false smut resistant and susceptible rice varieties. *BMC Genom*. 16:955. 10.1186/s12864-015-2193-x 26573512 PMC4647755

[B18] Hiller E. Heine S. Brunner H. Ripp S. (2007). Candida albicans Sun41p, a putative glycosidase, is involved in morphogenesis, cell wall biogenesis, and biofilm formation. *Eukaryotic Cell.* 6 2056–2065. 10.1128/EC.00285-07 17905924 PMC2168408

[B19] Kandawatte T. C. Peng J. Li X. Xing Q. Liu M. Zhang W. (2020). LtEPG1, a secretory endopolygalacturonase protein, regulates the virulence of *Lasiodiplodia theobromae* in *Vitis vinifera* and is recognized as a microbe-associated molecular patterns. *Phytopathology* 110 1727–1736. 10.1094/PHYTO-04-20-0118-R 32460690

[B20] Kim D. Langmead B. Salzberg S. (2015). HISAT: a fast spliced aligner with low memory requirements. *Nat. Methods* 12 357–360. 10.1038/nmeth.3317 25751142 PMC4655817

[B21] Kubicek C. P. Starr T. L. Glass N. L. (2014). Plant cell wall-degrading enzymes and their secretion in plant-pathogenic fungi. *Annu. Rev. Phytopathol.* 52, 427–451. 10.1146/annurev-phyto-102313-045831 25001456

[B22] Kuzentsov E. Kučerová H. Váchová L. Palková Z. (2013). SUN family proteins Sun4p, Uth1p and Sim1p are secreted from *Saccharomyces cerevisiae* and produced dependently on oxygen level. *PLoS One* 8:e73882. 10.1371/journal.pone.0073882 24040106 PMC3770667

[B23] Li B. Dewey C. (2011). RSEM: accurate transcript quantification from RNA-Seq data with or without a reference genome. *BMC Bioinform.* 12:323. 10.1186/1471-2105-12-323 21816040 PMC3163565

[B24] Li P. Gu Y. Li J. Xie L. Li X. Xie J. (2017). Mycobacterium tuberculosis major facilitator superfamily transporters. *J. Membr. Biol.* 250 573–585. 10.1007/s00232-017-9982-x 28852815

[B25] Li Y. Wang M. Liu Z. Zhang K. Cui F. Sun W. (2019). Towards understanding the biosynthetic pathway for ustilaginoidin mycotoxins in *Ustilaginoidea virens*. *Environ. Microbiol.* 21 2629–2643. 10.1111/1462-2920.14572 30807673

[B26] Liang Y. Yu H. Wang C. Cong J. Xu J. (2018). Targeted deletion of the USTA and UvSLT2 genes efficiently in *Ustilaginoidea virens* with the CRISPR-Cas9 system. *Front. Plant Sci.* 9:699. 10.3389/fpls.2018.00699 29881395 PMC5976777

[B27] Lin H. C. Yu P. L. Chen L. H. Tsai H. C. Chung K. R. (2018). A major facilitator superfamily transporter regulated by the stress-responsive transcription factor Yap1 is required for resistance to fungicides, Xenobiotics, and Oxidants and full virulence in *Alternaria alternata*. *Front. Microbiol.* 9:2229. 10.3389/fmicb.2018.02229 30279684 PMC6153361

[B28] Love M. Huber W. Anders S. (2014). Moderated estimation of fold change and dispersion for RNA-seq data with DESeq2. *Genome Biol.* 15:550. 10.1186/s13059-014-0550-8 25516281 PMC4302049

[B29] Lv B. Zheng L. Liu H. Tang J. Hsiang T. Huang J. (2016). Use of random T-DNA mutagenesis in identification of gene UvPRO1, A regulator of conidiation, stress response, and virulence in Ustilaginoidea virens. *Front. Microbiol.* 7:2086. 10.3389/fmicb.2016.02086 28082958 PMC5186764

[B30] Martins M. P. Silva L. G. Rossi A. Sanches P. R. Souza L. D. R. Martinez-Rossi N. M. (2019). Global analysis of cell wall genes revealed putative virulence factors in the dermatophyte *Trichophyton rubrum*. *Front. Microbiol.* 10:2168. 10.3389/fmicb.2019.02168 31608026 PMC6761320

[B31] Meng S. Xiong M. Jagernath J. S. Wang C. Qiu J. Shi H. (2020). UvAtg8-mediated autophagy regulates fungal growth, stress responses, conidiation, and pathogenesis in *Ustilaginoidea virens*. *Rice* 13:56. 10.1186/s12284-020-00418-z 32785866 PMC7423828

[B32] Millet N. Moya-Nilges M. Sachse M. Locker J. K. Latgé J.-P. Mouyna I. (2019). *Aspergillus fumigatus* exoβ(1-3)glucanases family GH55 are essential for conidial cell wall morphogenesis. *Cell Microbiol.* 21:e13102. 10.1111/cmi.13102 31424155

[B33] Mouyna I. Aimanianda V. Hartl L. Prevost M. C. Sismeiro O. Dillies M. A. (2016). GH16 and GH81 family β-(1,3)-glucanases in *Aspergillus fumigatus* are essential for conidial cell wall morphogenesis. *Cell Microbiol.* 18 1285–1293. 10.1111/cmi.12630 27306610

[B34] Norice C. T. Smith J. F. J. Solis N. Filler S. G. Mitchell A. P. (2007). Requirement for *Candida albicans* SUN41 in biofilm formation and virulence. *Eukaryotic Cell.* 6 2046–2055. 10.1128/EC.00314-07 17873081 PMC2168420

[B35] Pérez-Hernández A. González M. González C. van Kan J. A. L. Brito N. (2017). BcSUN1, a B. cinerea SUN-family protein, is involved in virulence. *Front. Microbiol.* 8:35. 10.3389/fmicb.2017.00035 28163701 PMC5247446

[B36] Pertea M. Pertea G. Antonescu C. Chang T.-C. Mendell J. Salzberg S. (2015). StringTie enables improved reconstruction of a transcriptome from RNA-seq reads. *Nat. Biotechnol.* 33 290–295. 10.1038/nbt.3122 25690850 PMC4643835

[B37] Plaza V. Silva-Moreno E. Castillo L. (2020). Breakpoint: cell wall and glycoproteins and their crucial role in the *Phytopathogenic* fungi infection. *Curr. Protein Pept. Sci.* 21:227. 10.2174/1389203720666190906165111 31490745

[B38] Ritch J. J. Davidson S. M. Sheehan J. J. Austriaco N. (2010). The Saccharomyces SUN gene, UTH1, is involved in cell wall biogenesis. *FEMS Yeast Res.* 10 168–176. 10.1111/j.1567-1364.2009.00601.x 20070376 PMC2852170

[B39] Sorgo A. G. Heilmann C. J. Brul S. Koster C. G. D. Klis F. M. (2013). Beyond the wall: Candida albicans secret(e) s to survive. *FEMS Microbial. Lett.* 338 10–17. 10.1111/1574-6968.12049 23170918

[B40] Sun W. X. Fan J. Fang A. F. Li Y. Wang W. M. (2020). Ustilaginoidea virens: insights into an emerging rice pathogen. *Annu. Rev. Phytopathol.* 58 363–385. 10.1146/annurev-phyto-010820-012908 32364825

[B41] Tang J. Bai J. Chen X. Zheng L. Liu H. Huang J. (2019). Two protein kinases UvPmk1 and UvCDC2 with signifcant functions in conidiation, stress response and pathogenicity of rice false smut fungus *Ustilaginoidea virens*. *Curr. Genet.* 66 409–420. 10.1007/s00294-019-01029-y 31489464

[B42] Tang Y. X. Jin J. Hu D. W. Yong M. L. Xu Y. He L. P. (2013). Elucidation of the infection process of *Ustilaginoidea virens* (cooke) tak. (*Teleomorph: Villosiclava virens*) in rice spikelets. *Plant Pathol.* 62 1–8. 10.1111/j.1365-3059.2012.02629.x

[B43] Wang S. C. Davejan P. Hendargo K. J. Javadi-Razaz I. Chou A. Yee D. C. (2020). Expansion of the major facilitator superfamily (MFS) to include novel transporters as well as transmembrane-acting enzymes. *Biochim. Biophys. Acta Biomembr.* 1862:183277. 10.1016/j.bbamem.2020.183277 32205149 PMC7939043

[B44] Wu Z. Wang S. Yuan Y. Zhang T. Liu J. Liu D. (2016). A novel major facilitator superfamily transporter in *Penicillium digitatum* (PdMFS2) is required for prochloraz resistance, conidiation and full virulence. *Biotechnol. Lett.* 38 1349–1357. 10.1007/s10529-016-2113-4 27146209

[B45] Xie C. Mao X. Huang J. Ding Y. Wu J. Dong S. (2011). KOBAS 2.0: a web server for annotation and identification of enriched pathways and diseases. *Nucleic Acids Res.* 39 W316–W322. 10.1093/nar/gkr483 21715386 PMC3125809

[B46] Xie S. Wang Y. Wei W. Li C. Liu Y. Qu J. (2019). The *Bax inhibitor* UvBI-1, a negative regulator of mycelial growth and conidiation, mediates stress response and is critical for pathogenicity of the rice false smut fungus *Ustilaginoidea virens*. *Curr. Genet.* 65 1185–1197. 10.1007/s00294-019-00970-2 30993412

[B47] Xiong M. Meng S. Qiu J. Shi H. Shen X. Kou Y. (2020). Putative phosphatase UvPsr1 is required for mycelial growth, conidiation, stress response and pathogenicity in *Ustilaginonidea virens*. *Rice Sci.* 27 529–536. 10.1016/j.rsci.2020.09.009

[B48] Yong M. Yu J. Pan X. Yu M. Cao H. Qi Z. (2020). MAT1-1-3, a mating type gene in the *Villosiclava virens*, is required for fruiting bodies and sclerotia formation, asexual development and pathogenicity. *Front. Microbiol.* 11:1337. 10.3389/fmicb.2020.01337 32714294 PMC7344243

[B49] Yu J. Yu M. Song T. Cao H. Pan X. Yong M. (2019). A homeobox transcription factor UvHOX2 regulates chlamydospore formation, conidiogenesis, and pathogenicity in *Ustilaginoidea virens*. *Front. Microbiol.* 10:1071. 10.3389/fmicb.2019.01071 31281290 PMC6596325

[B50] Yu M. Yu J. Cao H. Yong M. Liu Y. (2019). Genome-wide identification and analysis of the gata transcription factor gene family in *Ustilaginoidea virens*. *Genome* 62 807–816. 10.1139/gen-2018-0190 31437416

[B51] Yu M. Yu J. Hu J. Huang L. Wang Y. Yin X. (2015). Identification of pathogenicity-related genes in the rice pathogen *Ustilaginoidea virens* through random insertional mutagenesis. *Fungal. Genet. Biol.* 76 10–19. 10.1016/j.fgb.2015.01.004 25636735

[B52] Zhang N. Yang J. Fang A. Wang J. Li D. Li Y. (2020). The essential effector SCRE1 in *Ustilaginoidea virens* suppresses rice immunity via a small peptide region. *Mol. Plant Pathol.* 21 445–459. 10.1111/mpp.12894 32087618 PMC7060142

[B53] Zhang Y. Zhang K. Fang A. Han Y. Yang J. Xue M. (2014). Specific adaptation of *Ustilaginoidea virens* in occupying host florets revealed by comparative and functional genomics. *Nat. Comm.* 5:3849. 10.1038/ncomms4849 24846013

[B54] Zheng D. Wang Y. Han Y. Xu J. R. Wang C. (2016). UvHOG1 is important for hyphal growth and stress responses in the rice false smut fungus *Ustilaginoidea virens*. *Sci. Rep.* 6:24824. 10.1038/srep24824 27095476 PMC4837404

