# Supplementary material for: SUN-Family Protein UvSUN1 Regulates the Development and Virulence of Ustilaginoidea virens
Source: Front Microbiol. 2021 Sep 13;12:739453. doi: 10.3389/fmicb.2021.739453 (PMC8473917; doi:10.3389/fmicb.2021.739453)
Supplement: Supplementary file 1 [file Data_Sheet_1.PDF]

Supplementary Figures and Legends

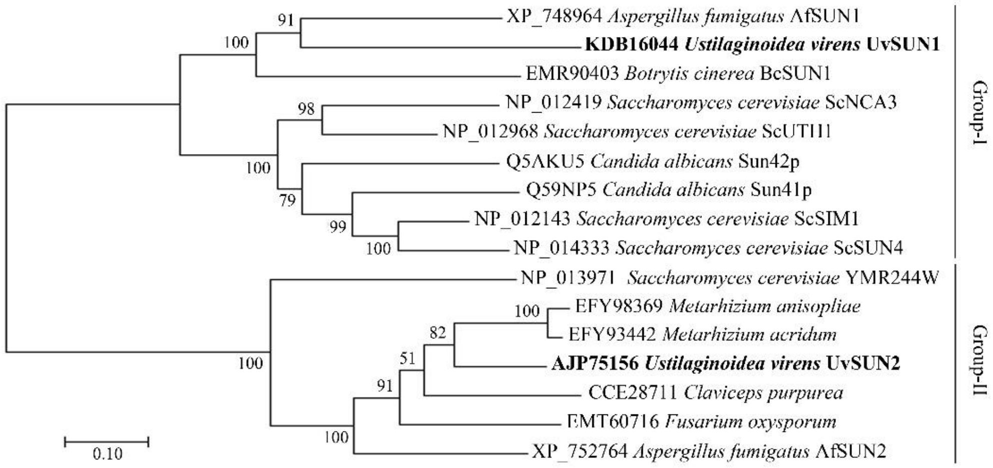

Supplementary Figure S1. Phylogeny of Sun proteins identified in yeast and filamentous ascomycetes. The sequences of known and putative Sun proteins identified in the deduced proteomes were aligned and an unrooted tree was generated by neighbor-joining analysis with 1000 bootstrap replicates (MEGA 7.0). Numbers on the branches represent the percentage of replicates supporting each branch. Two main groups were defined: Group I contains typical Sun proteins including *S. cerevisiae* Sim1p, Uth1p, Nac3p and Sun4p, while Group II contains atypical Sun proteins including *S. cerevisiae* YMR244W. Labels on the right indicate the accession number in the GenBank database and the fungal species.

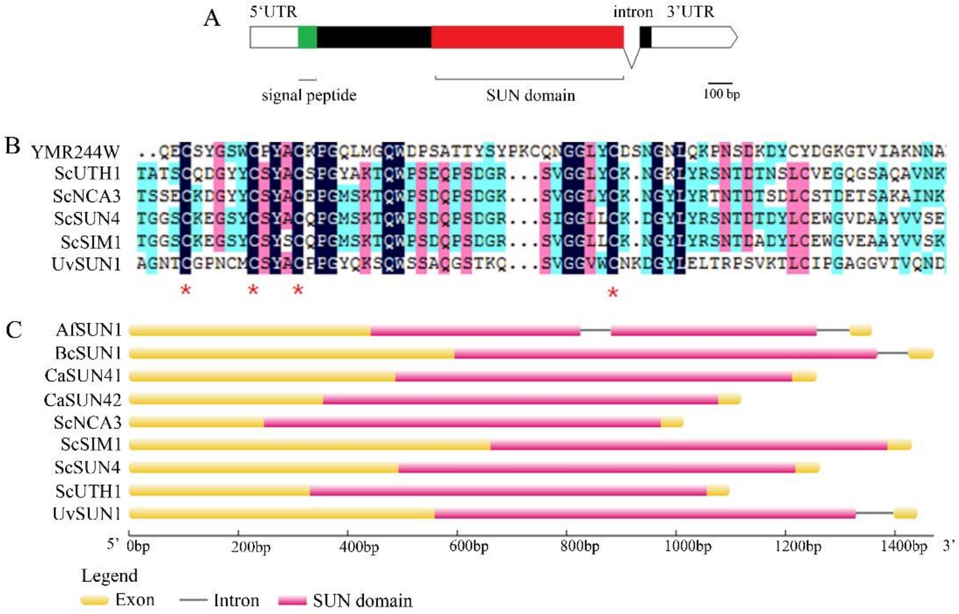

Supplementary Figure S2. Analysis of schematic diagram and conserved motif of *Uvsun1* with members of SUN protein family from various fungi. (A) Schematic diagram of gene *Uvsun1*. The white box on both sides represents UTR region of the gene, the green box represents predicted protein signal peptide coding region, the red box represents predicted protein sun domain coding region, and the black box represents predicted protein coding region without known functional domain. (B) The amino acid sequences of conserved motifs between UvSUN1 and homologous proteins in *Saccharomyces cerevisiae* S288C were analyzed. The pink shade indicated that the amino acid identity of different proteins was 100%. The blue shade indicates that the amino acid identity of different proteins is 75-100%. The gray shade indicates that the amino acid identity of different proteins is 50-75%. (C) The distribution of SUN domain in genes. The yellow thick line indicates the exon, the black thin line indicates the intron, and the pink area indicates the position of SUN domain in the genes. Sc, Ca, Af and Bc represent *Saccharomyces cerevisiae* S288C, *Candida albicans* SC5314, *Aspergillus fumigatus* var. RP-2014 and *Botrytis cinerea* BcDW1, respectively.

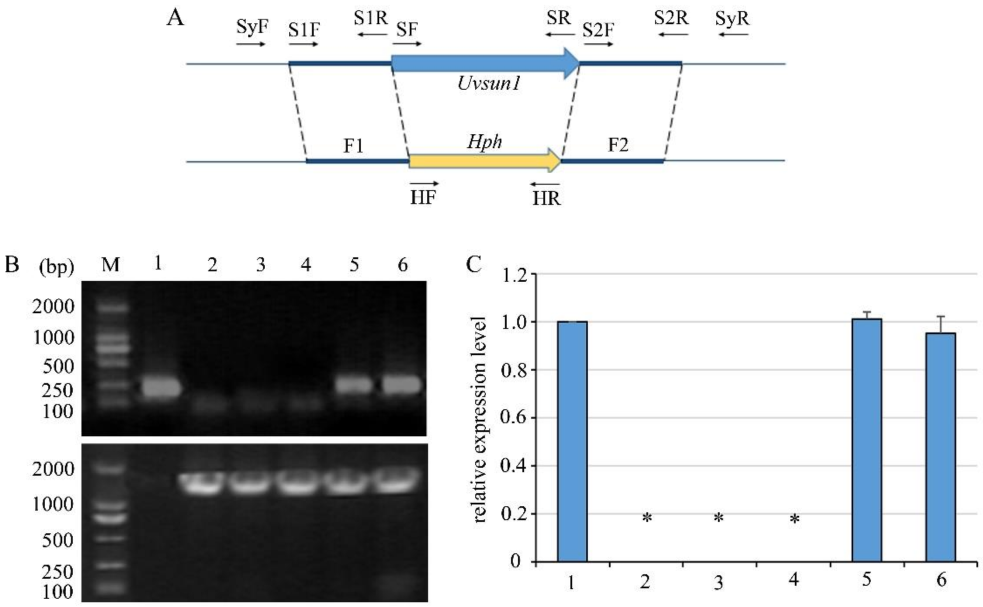

Supplementary Figure S3. *Uvsun1* was replaced with *Hph* using CRISPR/Cas9 technology. (A) The schematic diagram for *Uvsun1* gene replacement using the CRISPR/Cas9 system. The *Uvsun1* coding region in wild-type *U. virens* P1 was replaced with *Hph* (*hygromycin B-resistance*) gene to produce *Uvsun1* mutants. Primer pairs S1F and S1R for upstream of the gene, S2F and S2R for downstream of the gene, HF and HR for hygromycin resistance gene amplification. Primer pairs SF/SR, SyF/HR and SyR/HF were used for *Uvsun1* mutants screening. (B) The knockout mutants and complemented strains of *UvSUN1* gene were detected by PCR. The expression of *UvSUN1* gene was detected by primer pair qUvSUN1F and qUvSUN1R (up). The target fragment was amplified in the gene knockout mutants, but positive in P1 and complemented strains, and the length of PCR product met the expectation. Using HF and HR primers to amplify the *Hph* gene (down), the target fragment was not detected in P1. In the knockout and complemented mutants, the detection results were positive, and the length of PCR was in line with the expectation. (C) The expression of *UvUSN1* gene was detected by RT-PCR. The results showed that *UvUSN1* gene was expressed in P1 and complemented strains, but not in knockout mutants. 1, P1; 2, *UvSUN1-44*; 3, *UvSUN1-33*; 4, *UvSUN1-48*; 5, UvSUN1c1; 6, UvSUN1c2. Asterisks indicate significant differences (one-way ANOVA, *$p < 0.05$).

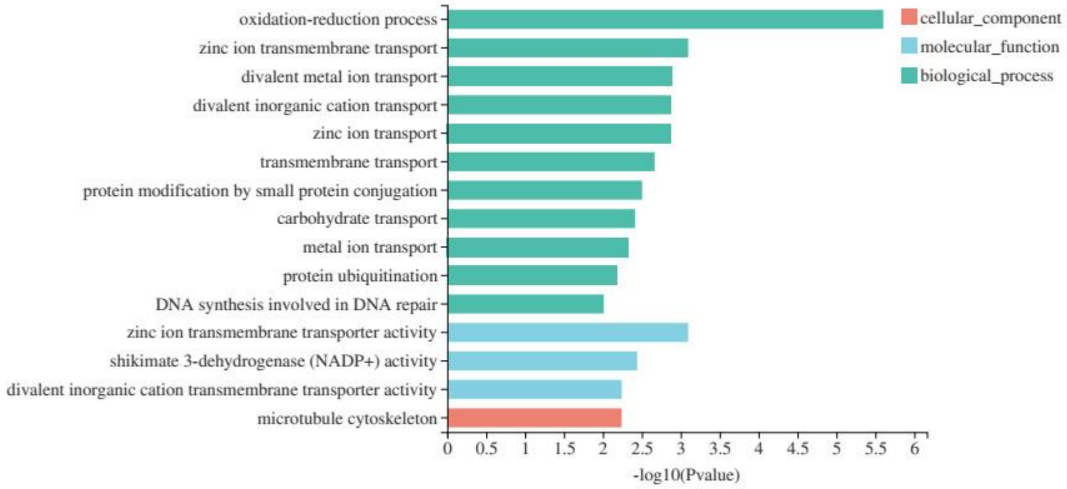

Supplementary Figure S4. Gene ontology (GO) enrichment analysis of significantly differentially expressed genes in △*Uvsun1* compared with WT. The *x*-axis indicates enrichment scores [–log10 (*p*-value)] (the *P*-value indicates the possibility of significant enrichment) for each GO item on the *y*-axis. The fold enrichment was calculated based on the frequency of genes annotated to the term compared to their frequency in all transcripts detected.

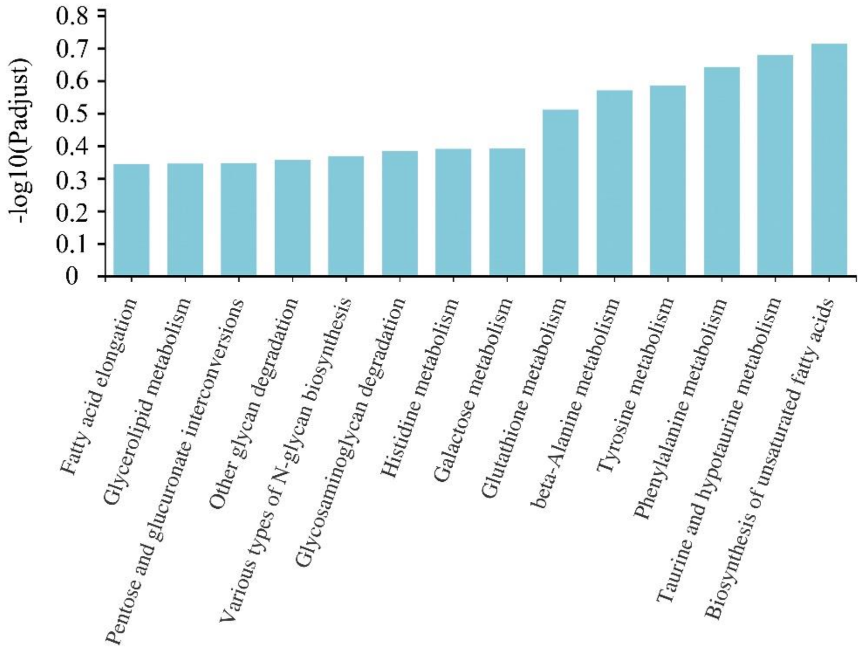

Supplementary Figure S5. Kyoto Encyclopedia of Genes and Genomes (KEGG) enrichment analysis of significantly differentially expressed genes in △*Uvsun1* compared with WT. The *y*-axis indicates enrichment scores [–log10 (*p*-value)] (the *P*-value indicates the possibility of significant enrichment) for each KEGG item on the *x*-axis. The fold enrichment was calculated based on the frequency of genes annotated to the term compared to their frequency in all transcripts detected.
