# Supplementary material for: SUN-Family Protein UvSUN1 Regulates the Development and Virulence of Ustilaginoidea virens
Source: Front Microbiol. 2021 Sep 13;12:739453. doi: 10.3389/fmicb.2021.739453 (PMC8473917; doi:10.3389/fmicb.2021.739453)
Supplement: Supplementary file 2 [file Table_1.DOCX]

**Supplementary Tables**

**Supplementary Table S1**

| Primers | Sequences (5’ - 3’) |
| --- | --- |
| S1F | TCGGTGGCCAGGGGTTTGTA |
| S1R | GCAGTTGGTCCCTTGGCATC |
| S2F | GGGAAGGGACATGGCTGTTA |
| S2R | GCAGTTGGTCCCTTGGCATC |
| HF | ACAGAAGATGATATTGAAGGAGC |
| HR | TACTCTATTCCTTTGCCCTCG |
| SF | atgaggaatatcattcaaatt |
| SR | ctagtagaagcgaatcacagc |
| SyF | AGAGGATGAAGGAGCAGAAC |
| SyR | ATGTCGGTTGGAGTTTGG |
| qSUN1F | CGGACGGTATCACATACATT |
| qSUN1R | AAGCGAATCACAGCCTTG |
| pKO1-UvSUN1 F | TTCTGACCCGGGGATCCGCCGATACAACTATCTAGCTT |
| pKO1-UvSUN1 R | GGCCAGTGCCAAGCTTCTCTCGGCATGTTATACTTGG |
| UvSUN1 Cr-F | ACCTgcagtgctagacgaagacgt |
| UvSUN1 Cr-R | AAACacgtcttcgtctagcactgc |
| 1132qF | TCAAGCCTCCAAACGACCACT |
| 1132qR | CCCTTGAGCTTGGCACCGAT |
| 3597qF | GTCCTGCAAGCTGCTCAAGCC |
| 3597qR | AGATTGCCCAGCCCCTTGACC |
| 2282qF | GCCGAAGCGTTCAACAATGTCCA |
| 2282qR | AGCCCTTGTCGGATTTGCTCT |
| 2474qF | GGTTCGCATCAAGACCATTGCC |
| 2474qR | ACACATTCTCAACCGTCAGCTC |
| 1061qF | GCCCGTGGAAACAACCCCTG |
| 1061qR | CCCCAGCCGTTCCTGTCGTC |
| 5043qF | TACAAGTCGAAGCCAATCCCT |
| 5043qR | CCAACTGTTTCAGAGCCTCGT |
| 1542qF | CGTCCACCGCGACCTCAAGCTG |
| 1542qR | CCAAGCTCCAGACGTCGACCT |
| 2973qF | ACACCCTTTTCCCCACGCTTG |
| 2973qR | TCTCGTCGCTCCGCTCTTCGTT |
| 6245qF | AACAAAAGATTGACTTTGCCAT |
| 6245qR | AAATCCATTCTTTGGCGCAAC |
| 2134qF | TTCCTCAGGCTTTCGTACACA |
| 2134qR | GTTCACCGTGTAGACCATGC |
| 7554qF | ACGAGCCTATACAACATCGGAA |
| 7554qR | AGAAATGCGGGCAAATACCAC |
| 6673qF | ATGTTGGCACCTGGCTTCACCC |
| 6673qR | AGGTTGTCACCGCCCTTACCAC |
| 2091qF | TCGAAAGCTGCCTTGGACCC |
| 2091qR | ACTTCTCCGTGGCCGTTCAACA |
| 8243qF | ACAACCCGTGGCGCTTCTACCAG |
| 8243qR | AGCGCCACCTTGACCTCGTT |
| 7112qF | CCGCCCCATTCCTACGACGAG |
| 7112qR | TCGCCACTCCATTCAAACGGGAC |
| *β-*tubulin F | AGGTTGCGTTGAAGGAGGTT |
| *β-*tubulin R | GAGGTGGAGTTGCCGATAAA |
